# Human adenovirus type 3 restores pharmacologically inhibited exosomal cargo in lung carcinoma cells

**DOI:** 10.3389/fphar.2024.1339862

**Published:** 2024-02-21

**Authors:** Ayodeji O. Ipinmoroti, Rachana Pandit, Brennetta J. Crenshaw, Brian Sims, Qiana L. Matthews

**Affiliations:** ^1^ Microbiology Program, Alabama State University, Montgomery, AL, United States; ^2^ Departments of Pediatrics and Cell, Developmental and Integrative Biology, Division of Neonatology, University of Alabama at Birmingham, Birmingham, AL, United States; ^3^ Department of Biological Sciences, College of Science, Technology, Engineering, and Mathematics, Alabama State University, Montgomery, AL, United States

**Keywords:** inhibitors, HAdV3, exosomes, cargo, Climbazole, Heparin, drug repurposing

## Abstract

**Introduction:** Drug repurposing is fast growing and becoming an attractive approach for identifying novel targets, such as exosomes for cancer and antiviral therapy. Exosomes are a specialized class of extracellular vesicles that serve as functional mediators in intercellular communication and signaling that are important in normal physiological functions. A continuously growing body of evidence has established a correlation between the abnormal release of exosomes with various viral disease pathologies including cancer. Cells that are virus-infected release exosomes known to influence the process via the loading and transfer of viral components, such as miRNA, small (s) RNA, DNA, and proteins. Inhibition of exosome release may abate the spread and severity of viral infection, thus making exosomes an attractive target for antiviral therapies. We previously demonstrated the pharmacological inhibition of exosomes.

**Methods:** Herein, we used a cell-based assay to determine the effect of Human adenovirus type 3 (HAdV3) on the exosome inhibition process by azole and Heparin derivatives. HAdV3-infected cells were treated with two concentrations of each inhibitor at different time points.

**Results:** HAdV3 activities led to increased total sRNA, DNA, and exosome particle concentrations via particle tracking in the presence of Climbazole and Heparin relative to uninfected exosomes. In addition, there was an increased expression of classical markers such as ALG-2 interacting protein X (ALIX), and tetraspanin (CD63), (*p* < 0.05) and upregulated transcription factor interferon regulatory factor (IRF) 8 in the presence of HAdV3 after 24 hours (h) of treatment. Whereas higher concentrations of Climbazole and Heparin sodium salt were found to inhibit total exosome protein (*p* < 0.001) and exo-RNA (*p* < 0.01) content even in the presence of HAdV3 relative to infected exosomes only. Activities of HAdV3 in the presence of selected inhibitors resulted in the positive regulation of exosome related DNA damage/repair signaling proteins. Blocking exosome secretion partially obstructed viral entry. Immunological studies revealed that HAdV3 fiber protein levels in A549 cells were reduced at all concentrations of Climbazole and Heparin and both multiplicities of infections (*p* < 0.001).

**Discussion:** Our findings suggest that while HAdV may bolster inhibited exosome content and release when modulating certain activities of the endosomal pathway mediators, HAdV entry might be constrained by the activities of these pharmacological agents.

## Introduction

Human adenoviruses (HAdVs), belong to the family Adenoviridae and the genus, *Mastadenovirus*. They are non-enveloped, icosahedral, double-stranded DNA viruses, and there are about 67 serovars that are known to cause severe infections, leading to pneumonitis, cystitis, conjunctivitis, gastroenteritis, and/or acute hemorrhagic cystitis. Human adenovirus type 3 (HAdV-3) is one of the most virulent of the HAdVs (Ipinmoroti et al., 2021). It causes respiratory infections globally, affecting both children and adults and resulting in significant morbidity and mortality. This impact is particularly pronounced in the pediatric age group, including neonates. HAdV-3 has emerged as a predominant agent of acute respiratory infections, contributing to 15%–87% of all adenoviral respiratory cases worldwide (Haque et al., 2018). Pulmonary infections with HAdV-3 strains can lead to complications such as bronchiectasis, bronchiolitis obliterans, unilateral hyperlucent lung, and persistent abnormalities in pulmonary function (Haque et al., 2018; Liu et al., 2020). Due to the infectious nature of these viruses, they could employ extracellular vesicle-assisted entry as a cellular mechanism of entry (Echavarría, 2008; Nemerow et al., 2012; Lion, 2014; Crenshaw et al., 2019a). Extracellular vesicles (EVs) released from all cell types contribute to intercellular communication including signaling and stimulation via horizontal transfer of biomolecules such as nucleic acids, proteins, and lipids. There are different classes of EVs out of which exosomes have proven to be the most versatile in both laboratory settings and clinical trials as a function of their role in the advancement or cessation of various pathological processes. Exosomes range between 30 and 150 nm (Liu et al., 2019; Ipinmoroti et al., 2021; Ipinmoroti et al., 2022). Exosomes released by virus-infected cells carry viral components, miRNAs, mRNAs, short and long non-coding RNA strands (lncRNA), DNA, carbohydrates, cytokines, and enzymes. Exosome-based treatment of some viruses may include inhibition of exosome release and uptake, amidst others like drug delivery, vaccines, and therapy (Kowal et al., 2014; Hassanpour et al., 2020). Studies carried out on exosomes and their impact on viral transmission, progression, and pathogenesis have revealed that during typical viral infection, viruses can induce profound alterations in the exosome developmental process. Exosome release during the virus-induced pathological condition can induce both protective and debilitative effects on the immune system (Zhang et al., 2020; Ipinmoroti et al., 2022). Exosomes isolated from respiratory syncytial virus-infected cells were shown to induce an innate immune response by activating cytokine and chemokine release from human monocytes and airway epithelial cells (Chahar et al., 2018; Alem et al., 2021). Exosomes derived from monocyte cells infected with the Rift Valley fever virus-induced IFN-β and autophagy activation resulted in resistance to subsequent infection (Alem et al., 2021). The protective role conferred by monocyte-derived exosomes was attributed to the induction of IFN-β which emphasizes the importance of exosomes in innate immune response (Alem et al., 2021). Regarding the consequences of virus-exosome interaction, the relationship has been proven to go both ways. While exosomes influence viral infection via viral protein trafficking and involvement in cell signaling, viruses have also been shown to hijack endosomal pathways to facilitate both entry and release with optimal influence on exosome release (Sims et al., 2018; Ipinmoroti and Matthews, 2020).

A recent hypothesis has reconciled a current paradigm involving a retroviruses-facilitated infection cycle involving an endosomal origin, a process that could enable them to evade natural adaptive immune response or the one provided by retroviral antigen vaccines (Gould et al., 2003; Sims et al., 2017). Blockage of exosome-mediated Human Immunodeficiency Virus-1 (HIV-1) entry/uptake in Human cluster of differentiation (CD4^+^) lymphoblastoid T cells by tetraspanin binding, also demonstrated the importance of virus-exosome interaction (Sims et al., 2018). Coxsackievirus B (CVB) similar to HAdV has been previously described to interact with coxsackie and adenovirus receptor (CAR) to gain entry into the host cell, a process that was described to be facilitated by exosome surface protein engaging target cell. This provides an alternative route of CVB infection followed by enhanced exosome function during the infection process (Fu and Xiong, 2023).

In this study, we explored one of the recent and fast-growing fields of pharmacology known as “*drug repurposing*,” which involved the use of pharmacological agents to induce biological processes other than their intended usage. It is well-established that exosomes are released by tumor cells, and their circulating levels are elevated in patients with cancer and other aggressive diseases. One of the cancer-derived exosome-associated surface receptors known as Heparan-Sulfate proteoglycan has been demonstrated to be relevant in exosome uptake by effectively inhibiting exosome-mediated tumorigenesis (Christianson et al., 2013). It was shown that Oral Squamous Cell Carcinoma (OSCC) treatment with Heparin inhibited exosome uptake by recipient cells including tumor growth induced by OSCC-derived exosomes (Sento et al., 2016). Another study revealed that Ketoconazole, an antifungal medication has been shown to inhibit pathways of exosomal biogenesis and secretion via altering the exosome concentrations, exosome biogenesis, trafficking pathways, and protein endoplasmic reticulum kinase (pERK) expression (Greenberg et al., 2021). Datta et al. identified another potent inhibitor of exosome development to Climbazole. The study reported that after screening 4580 compounds identified to be potential inhibitors of exosome biogenesis and release. Climbazole was found to be a potent inhibitor by demonstrating promising activities necessary for the depletion of exosomes in cancer therapy (Datta et al., 2018; Zhang and Yu, 2019).

Despite exosomes’ explicit role in disease pathogenesis, there has only been little advancement in targeting pathways involved in their biogenesis and release (Datta et al., 2018), and examining how certain pathological conditions like viral infection influence their development. This study was set to determine the augmenting effect of HAdV3 on exosome development and cargo trafficking in the presence of pharmacological inhibitory agents. Therefore, we focused on Climbazole and Heparin as clinically applicable drugs and explored whether HAdV3 could restore exosome development in the presence of Climbazole and Heparin as exosome inhibitors. We observed that both RNA and DNA concentrations were positively regulated in inhibitor-treated cell-derived exosomes in the presence of HAdV3. Exosome particle concentration, classical cargo and markers from virus-inhibitor-treated cells were induced above the uninfected cell-derived exosomes. There was also a positive correlation between virus protein expression and augmented levels of exosome credentials. These outcomes suggest that HAdV3 infection could induce exosome release and cargo loading in the presence of inhibitory agents. These findings reveal the possible restoring capacity of HAdV on exosome development and the augmenting impact it has on exosome-inhibited developmental pathways.

## Materials and methods

### Materials

#### Media and cell line

Dulbecco’s modified Eagle medium nutrient mixture/F-12 medium (DMEM-F12) containing L-glutamine, penicillin/streptomycin solution, and Corning regular fetal bovine serum (FBS) were purchased from (Fisher Scientific, Grand Island, NY, USA), Penicillin/Streptomycin (Pen-Strep) (Fisher Scientific, Grand Island, NY, USA), and Amphotericin-B were also obtained from (Fisher Scientific, Grand Island, NY, USA). Climbazole (Tokyo Chemical Industry, Tokyo, Japan) and Heparin sodium salt (EMD Millipore Corporation, Burlington, MA, USA) were the inhibitors used in this study. Unless otherwise indicated, all other drugs were purchased from commercial sources. A549 cells were used in this study. A549 cells were obtained from (American Type Culture Collection, Manassas, VA, USA). Cells were cultured in DMEM-F12 (Fisher Scientific, Grand Island, NY, USA) supplemented with 10% FBS, 1% Pen-Strep, and 0.2% Amphotericin-B (0.5 μg/mL). For virus infection, DMEM exosome-free media was prepared with exosome-depleted FBS using DMEM/F12 medium containing L-glutamine supplemented with 2% exosome-free Corning FBS, 1% Pen-Strep, and 0.2% Amphotericin-B (0.5 μg/mL) (Fisher Scientific, Grand Island, NY, USA). Cells were cultured at 37°C in a humidified atmosphere supplemented with 5% CO2.

#### Virus stocks

HAdV3 viral stocks used in this study were previously generated and stored at −80°C. Preparation of the final viral stock concentration was indexed at 2 × 10^9^ vp/μL.

### Experimental

#### Drug(s) and virus mix preparation in treatment of lung cells

**FIGURE 1:**
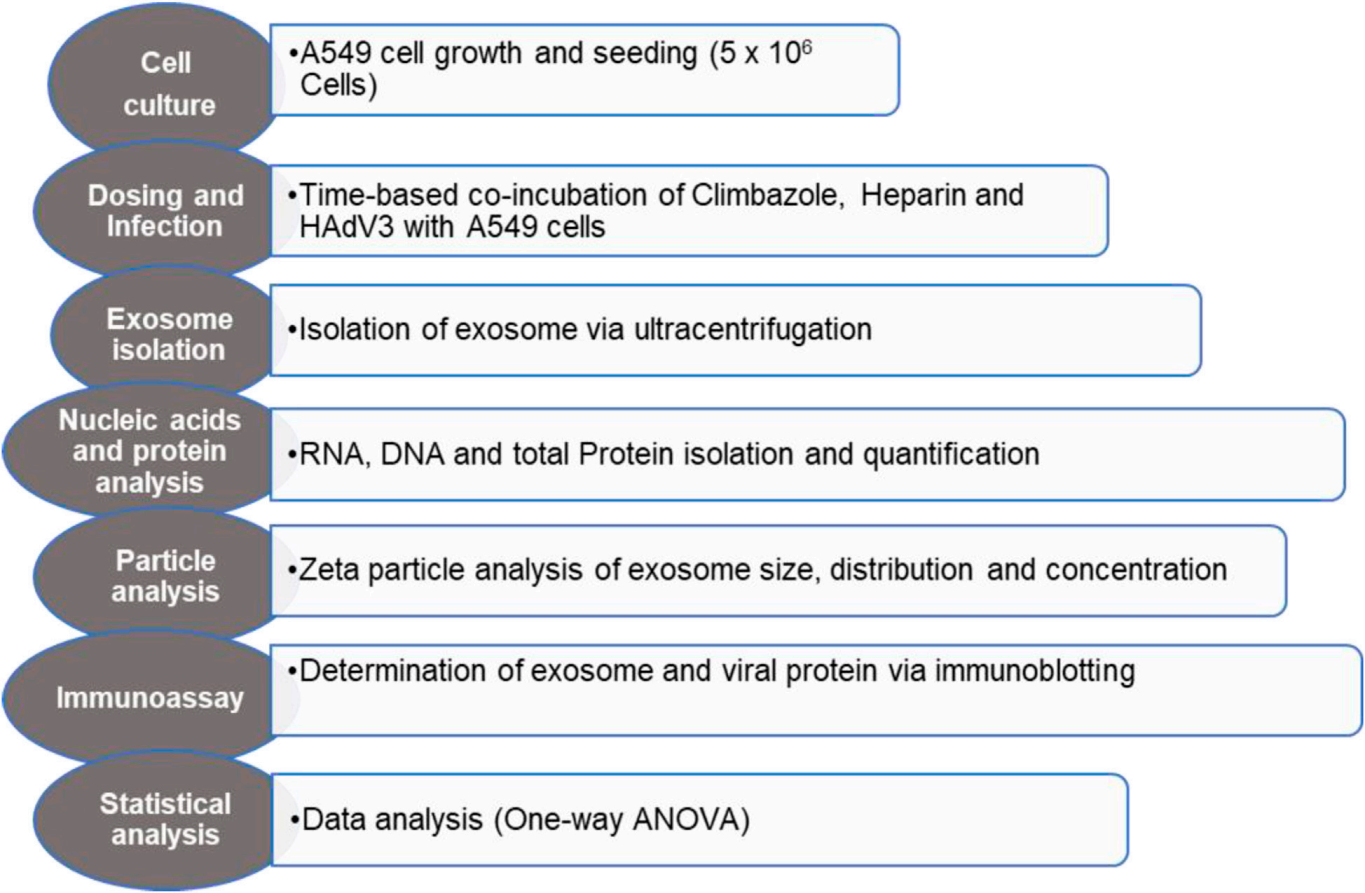
Graphical illustration of experimental workflow.

The effect of HAdV3 on exosome release from A549 cells in the presence of inhibitory drug(s) was determined, A549 cells are non-small cancer cells of the alveoli basal epithelial tissues. They have proven to be one of the most appropriate cell lines for virus-exosome study due to their high drug assimilation rate, vast yield of EVs/mL and receptive capacity for HAdV. Briefly, cells were trypsinized and counted a day before infection. Cell densities between 70% and 80% confluences at the time of infection were considered fit for the assay. Approximately 5x10^5^ cells were seeded in culture plates and incubated overnight at 37°C and 5% CO_2_ before treatment. This was followed by treatment with a prepared HAdV3-inhibitor mix consisting of predetermined concentrations of drug(s) (5 and 10 µM Climbazole, 0.176 and 0.88 µM Heparin) and HAdV3 particles (750 and 1500 multiplicities of infection (MOI);) for 6, 24 and 48 hour (h) (Workflow illustrated in Figure 1). Uninfected cells and HAdV3infected cells only served as controls. Plates were incubated for 6, 24, and 48 h, and each time point was evaluated as an independent experiment. The infected cell supernatant was collected and stored for EV isolation at the end of the incubation period.

#### Isolation and purification of exosomes from culture

EVs were isolated and purified from DMEM/F12 exosome-free cell culture media. In brief, EVs were isolated as previously described (Crenshaw et al., 2019b; Ipinmoroti et al., 2021). The media was collected after infection and spun down at 1,300 revolutions per minute (rpm) at 4°C for 10 minutes (min), using a Sorvall RT 6000 refrigerated centrifuge. The supernatant was collected, and the pellet was discarded. The collected supernatant was spun again at 3,900 rpm at 4°C for 10 min using a Sorvall RT 6000 refrigerated centrifuge and then filtered through a 10 mL syringe with a 25 mm syringe filter, with a porosity of 0.22 μM.

The volume of filtered supernatant was primed with PBS and centrifuged at 10,800 rpm for 45 min in an SW41T1 swinging bucket rotor at 4°C using a Beckman Coulter Optima L-70K Ultracentrifuge. The supernatant was collected and centrifuged at 32,000 rpm for 70 min in an SW41T1 swinging bucket rotor at 4°C using a Beckman Coulter Optima L-70K Ultracentrifuge. Approximately 500 *μ*L of purified EVs were collected below the meniscus of the centrifuge tube. Collected EVs were quantified using the Bradford–Lowry quantitation method (Sims et al., 2017; Sims et al., 2018; Crenshaw et al., 2019b; Kumar et al., 2020; Ipinmoroti et al., 2021).

#### Analysis of exosome particles by zeta-view particle tracking

To evaluate the size distribution and concentration of A549 cell-derived EVs (particle per mL), nanoparticle tracking analysis (NTA) was performed using NanoSight-LM10, Malvern Instrument, Inc., Malvern, UK. EV particle sizes were analyzed based on Brownian motion and light scattering. The samples were prepared at a dilution of 1:100 in PBS (1X) and loaded in a 0.3 mL disposable syringe. The NTA assesses particles based on the size and concentration of samples. The mean values of the replicate were recorded and processed for each reading frame of the five independent experiments (Sims et al., 2017; Sims et al., 2018; Crenshaw et al., 2019b; Kumar et al., 2020; Ipinmoroti et al., 2021).

#### Total exosomal RNA isolation and quantification

Exosomes were treated with Triton X-100 (1%) for 30 min followed by treatment with micrococcal nuclease (MNase) (5 μg/mL) containing 1 M tris HCl and 100 mM CaCl_2_ for 15 min. RNase inhibitor was added to the mix. Total RNAs were isolated by TRIzolTM LS reagent (Invitrogen, Waltham, MA, USA) protocol, and exosomal RNA (exoRNA) was resuspended in RNase/nuclease-free water. Isolated RNA was quantified, and the purity was assessed using a Nanodrop ONE^C^ instrument (Thermo Fisher Scientific Inc.).

#### Total DNA isolation and quantification

Exosomes (5 µg) were lysed and treated with deoxyribonuclease (DNase) I. DNA was isolated as a clean fraction using the TRIzol reagent protocol. Phenol-chloroform pelleted DNA was resuspended in 0.1 M sodium citrate (NA_3_C_6_H_5_O_7_) prepared in 10% ethanol and further purified using 75% ethanol. Isolated DNA was resuspended in nuclease-free water and quantity and purity were determined using the Nanodrop ONE^C^ instrument (Thermo Fisher Scientific Inc.).

#### Immunoblot Analysis

Expression of exosomal markers and content were evaluated via dot blot analysis. Briefly, 5 μg of EV protein or cell lysates were added to the reducing buffer in a 1:1 ratio and blotted on nitrocellulose membrane. The membrane was blocked for nonspecific binding and was incubated with the primary antibodies of CD63 (1: 500) (Santa Cruz Biotechnology, Dallas, Texas), cleaved caspase-1 (1: 500) (Fisher Scientific, Grand Island, NY, USA), Histone (H2A.x) (1: 500) (Cell Signaling Technology Inc., Danvers, MA, USA), toll like receptor (TLR) 7 (1: 500) (Abnova, Neihu District, Taipei City, Taiwan), Ras binding Protein (Rab) 35 (1: 500) (BioFisher Scientific, Rockford, IL, USA), tumor susceptibility gene (TSG)101 (1: 500) (Fisher Scientific, Grand Island, NY, USA), and ALIX (1: 500) (Fisher Scientific, Grand Island, NY, USA), overnight at 4°C. Proteins were further detected using Horseradish peroxidase-(HRP-) conjugated secondary antibodies (goat anti-rabbit (1: 500-1: 1,000) (Novus Biologicals LLC, Centennial, CO, USA) or goat anti-mouse (1: 500-1: 1,000) (Fisher Scientific, Grand Island, NY, USA)). Targeted protein signals were detected using Super-Signal West Femto Maximum Sensitivity Substrate (Invitrogen, MA, USA), and images were developed using Bio-Rad ChemiDoc™ XRS+ System (Bio-Rad Laboratories, Hercules, CA, USA) (Ipinmoroti et al., 2021).

#### HAdV fiber protein expression

Isolated exosome samples were added to the reducing buffer in a 4:1 ratio and boiled for 15 min at 95°C. Samples were loaded in a 4%–20% 1.5 mm Bio-Rad precast gel and allowed to migrate at 100 V. Proteins were transferred to a nitrocellulose membrane in a transfer chamber at 45 mA overnight. The membrane was blocked in 5% nonfat dry milk prepared in 0.2% Tween-20 and 1X Tris Buffered Saline (TBS) for 30-45 min at room temperature (RT). Primary antibodies against HAdV fiber (1: 250) were used in probing the membranes overnight at 4°C followed by secondary detection using HRP conjugated secondary antibody (1: 500-1: 2000). Signal was detected using SuperSignal West Femto Maximum Sensitivity Substrate (Thermo Fisher Scientific Inc, Rockford, IL, USA). The image was developed using Bio-Rad ChemiDoc XRS+ System (Bio-Rad Laboratories, Hercules, CA, USA).

#### Statistical analysis

Statistical analyses were performed using one-way analysis of variance (ANOVA) with Tukey *post hoc* analysis as the preferred test. Statistical significance is indicated by the mean ± standard deviation (SD) as follows: for multi-group comparisons, one-way ANOVA was used. Statistical significance was established to be *p < 0*.*05, **p < 0.01, ***p < 0.001, and ****p < 0.0001.

## Results

**FIGURE 2:**
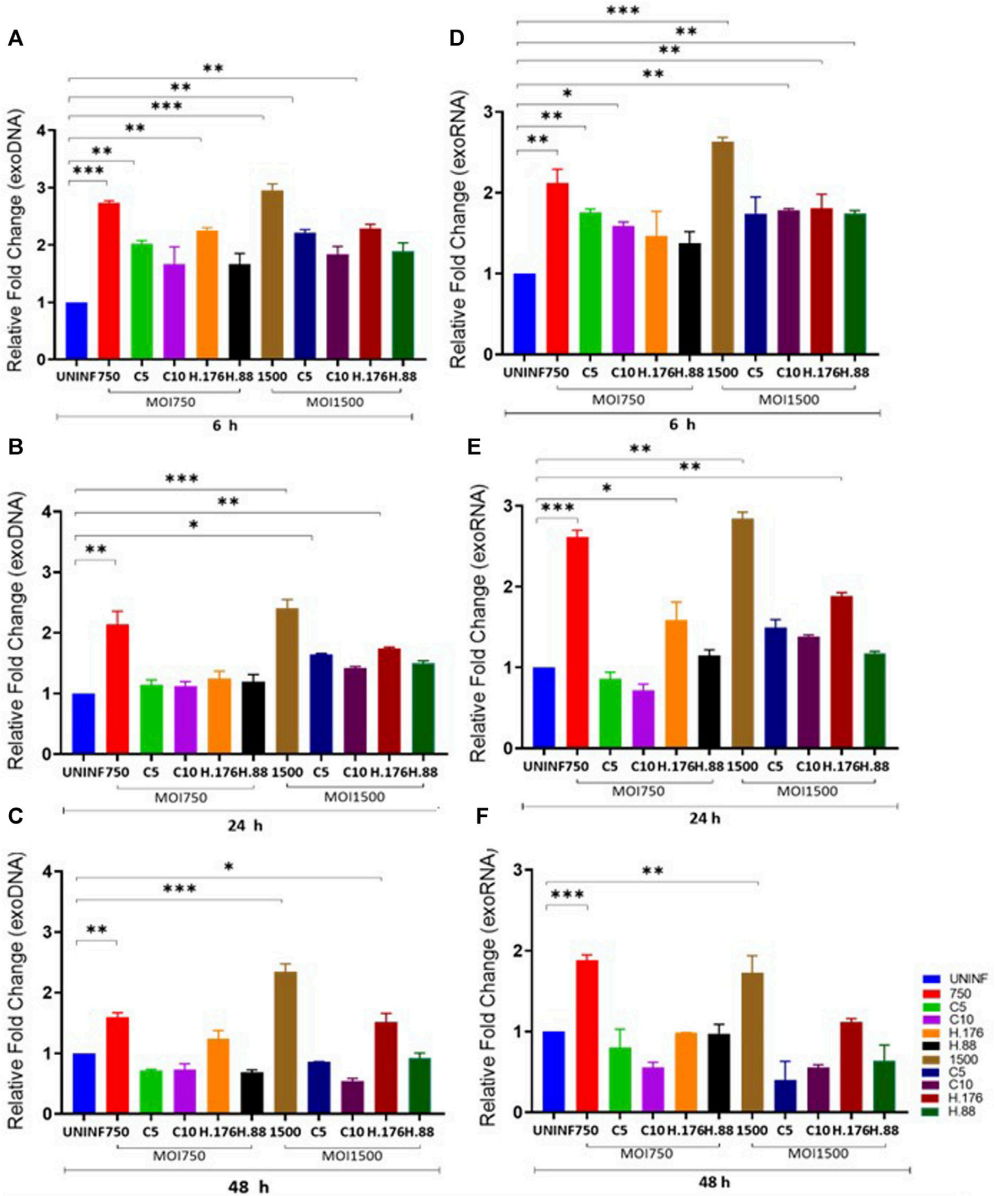
HAdV3 positively modulates exoDNA and RNA in the presence of Climbazole and Heparin. A549 cells were maintained overnight in exosome-free DMEM media and then treated with HAdV3-Climbazole, 5 µM (C5) and 10 µM (C10) or HAdV3-Heparin mixture 0.176 µM (H.176) and 0.88 µM (H.88) and MOIs (750 and 1500) of HAdV3 (*n* = 4). Infected cells only and uninfected cells (UNINF) served as control for exo RNA **(A)** 6, **(B)** 24, and **(C)** 48 h, and exoDNA at **(D)** 6, **(E)** 24, and **(F)** 48 h. ExoDNA showed a yield increase after 24 h HAdV3 infection in the presence of both Climbazole concentrations relative to uninfected cell-derived exoDNA, on the other hand, HAdV3 decreased exoRNA yield after 24 h of infection in the presence of Climbazole and Heparin. Mean values and SD were derived from four independent experiments. *Denotes significance at different levels of significance compared to controls and was calculated by one-way ANOVA using GraphPad Prism, Significance levels (*p < 0.05, *11 *0.01 and ***0.001).

Total exosomes containing DNA (exoDNA) and exosomes containing RNA (exoRNA) yield from inhibitor-treated cells were upregulated in the presence of HAdV3. EVs can be vehicles for the transfer and trafficking of short strands of DNA, sRNA, miRNA, and mRNAs. miRNAs are major players in the regulatory networks of gene expression via modulation by binding to complementary sequences of the mRNA of interest. Hundreds of genes can be targeted by only one miRNA highlighting their vital role in influencing the outcomes of gene regulation. Inhibition of certain mRNA has been linked to reduced severity of ischemia and neuroinflammatory/neurodegenerative diseases (Joshua et al., 2016). Comparative analysis of exosomal DNA (exoDNA) retrieved from patients with pancreatic ductal adenocarcinoma revealed that exoDNA carries a detectable level of *KRAS* mutation which makes them a candidate for liquid biopsy application (Allenson et al., 2017). We examined the effect of HAdV3 on exoDNA and exoRNA concentration in the presence of Climbazole and Heparin at different concentrations. HAdV3 augmented exoDNA levels in Climbazole (10 µM) and Heparin (0.88 µM) treated A549-exo (83%, and 70% respectively) (*p* < 0.001) at 6 h, but later decreased at 24 and 48 h (24%, and 30% respectively) (*p* < 0.01) relative to uninfected A549-exo (Figures 2A, B). The total DNA content of HAdV3-infected A549-exo was also boosted in Climbazole treatments at the early period of incubation but later declined after 48 h (Figure 2C). Total exoRNA concentration was significantly higher in A549-exo treated with HAdV3-Climbazole and Heparin complexes relative to HAdV3-infected A549-exo. Also, exoRNA content slightly declined after 24 and 48 h of treatment (Figures 2D–F). This finding indicates a decline in the HAdV3 effect on total DNA and RNA levels in the presence of Climbazole and Heparin as incubation time increases. Over time, the concentration of both RNA and DNA within exosomes decreased, potentially suggesting that the augmenting effect of HAdV3 may diminish or that the virus’s influence on exosome content changes over time. The most significant inference is that HAdV3 infection of lung cells appears to positively regulate the yield of inhibited exosome RNA and DNA contents. Thus, the presence of the virus may enhance the accumulation of exosomal nucleic acids, counteracting the inhibitory effects of Climbazole and Heparin observed initially. This suggests that HAdV3 infection of lung cells could positively regulate the yield of inhibited exosome DNA and RNA contents.

### Total exosome protein concentration

**FIGURE 3:**
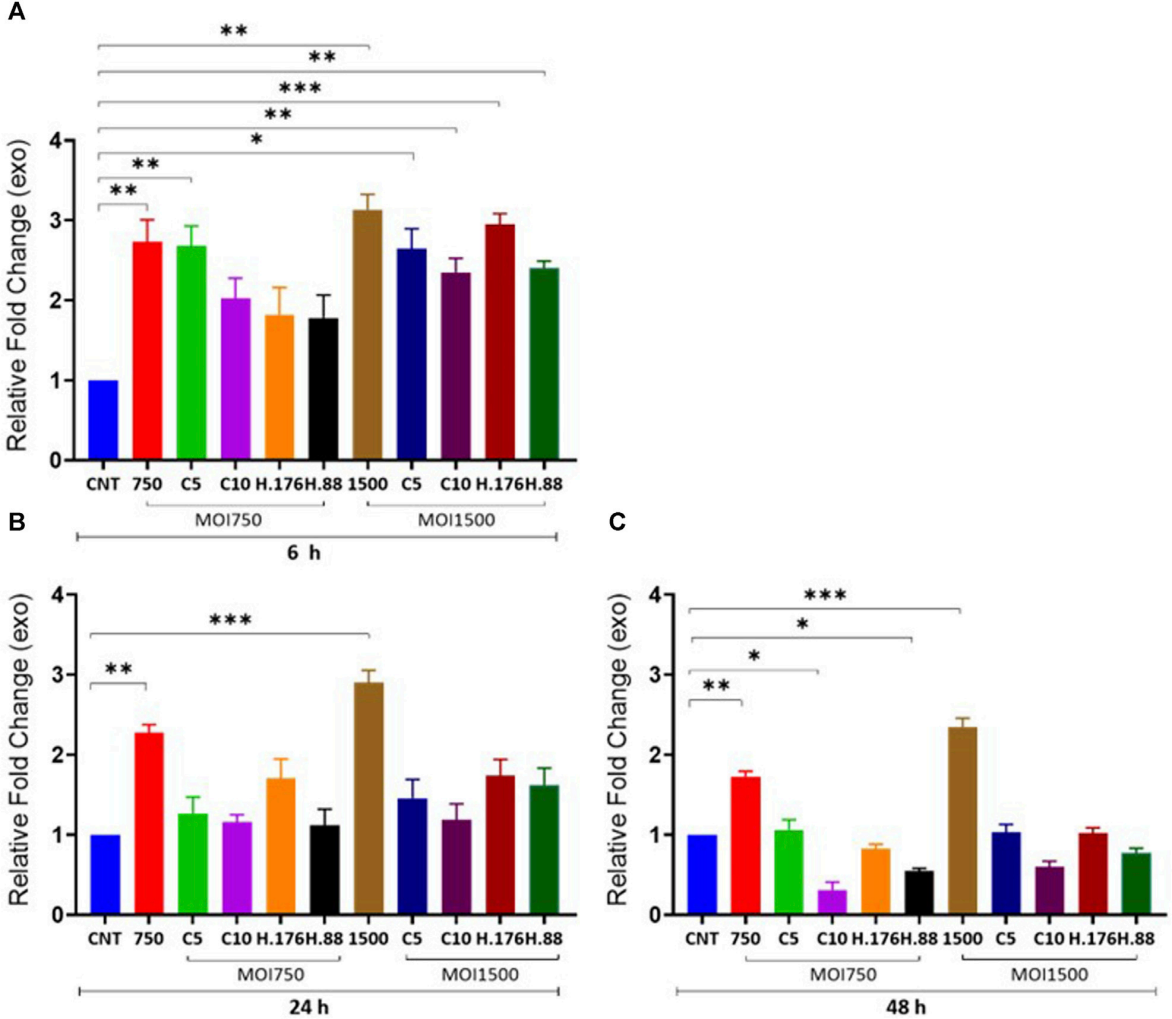
HAdV3 positively modulates total exosome protein in the presence of Climbazole and Heparin. A549 cells were maintained overnight in exosome-free DMEM media and then treated with HAdV3-Climbazole, 5 µM (C5) and 10 µM (C10) or HAdV3-Heparin mixture 0.176 µM (H.176) and 0.88 µM (H.88) and MOIs (750 and 1500) of HAdV3 (n = 4). Infected cells only and non-infected cells (CNT) served as control for **(A)** 6, **(B)** 24, and **(C)** 48 h. Total exosome protein derived from HAdV3 infected cells was augmented relative the control in the presence of both concentrations of Climbazole and Heparin but it decreased after 48 h of infection. Mean values and SD were derived from four independent experiments. *Denotes significance at different levels of significance compared to controls and was calculated by one-way ANOVA using GraphPad Prism, Significance levels (*p < 0.05, **0.01 and ***0.001).

We examined the effect of HAdV3 on exosomal protein concentration alongside corresponding cytoplasmic protein in the presence of inhibitors at different concentrations. We observed a significant decrease in total exosome protein concentration consistent with DNA findings. There was a significant increase in exosome protein release in HAdV3-treated exosomes in the presence of Climbazole and Heparin concentration (*p* < 0.01). Although total exosome protein levels were similar to the uninfected exosome after 24 and 48 h time points, however, there was a slight reduction after 48 h treatment when compared to the uninfected exosomes, suggesting that inhibitory capacities of Heparin and Climbazole were potent in the presence of HAdV3 after 48 h treatment (Figures 3A–C). In the presence of Climbazole and Heparin, there was a significant reduction in exosome protein release in HAdV3-treated exosomes. This implies that Climbazole and Heparin may have an inhibitory effect on the release of exosome proteins during HAdV3 infection. This inhibitory effect could be beneficial in certain contexts, such as reducing the spread of viral proteins via exosomes. The impact of Climbazole and Heparin on exosome protein release appears to be time-dependent. While the total exosome protein levels were similar to uninfected exosomes at 24 h, a slight reduction was observed after 48 h of treatment in the presence of HAdV3. This suggests that the inhibitory capacity of Climbazole and Heparin becomes more potent with time during HAdV3 infection. The finding that Climbazole and Heparin had a more pronounced effect on exosome protein release after 48 h suggests that these compounds may have a sustained inhibitory capacity over a longer duration of HAdV3 infection.

### Total particle concentration increased with HAdV3 treatment

**FIGURE 4:**
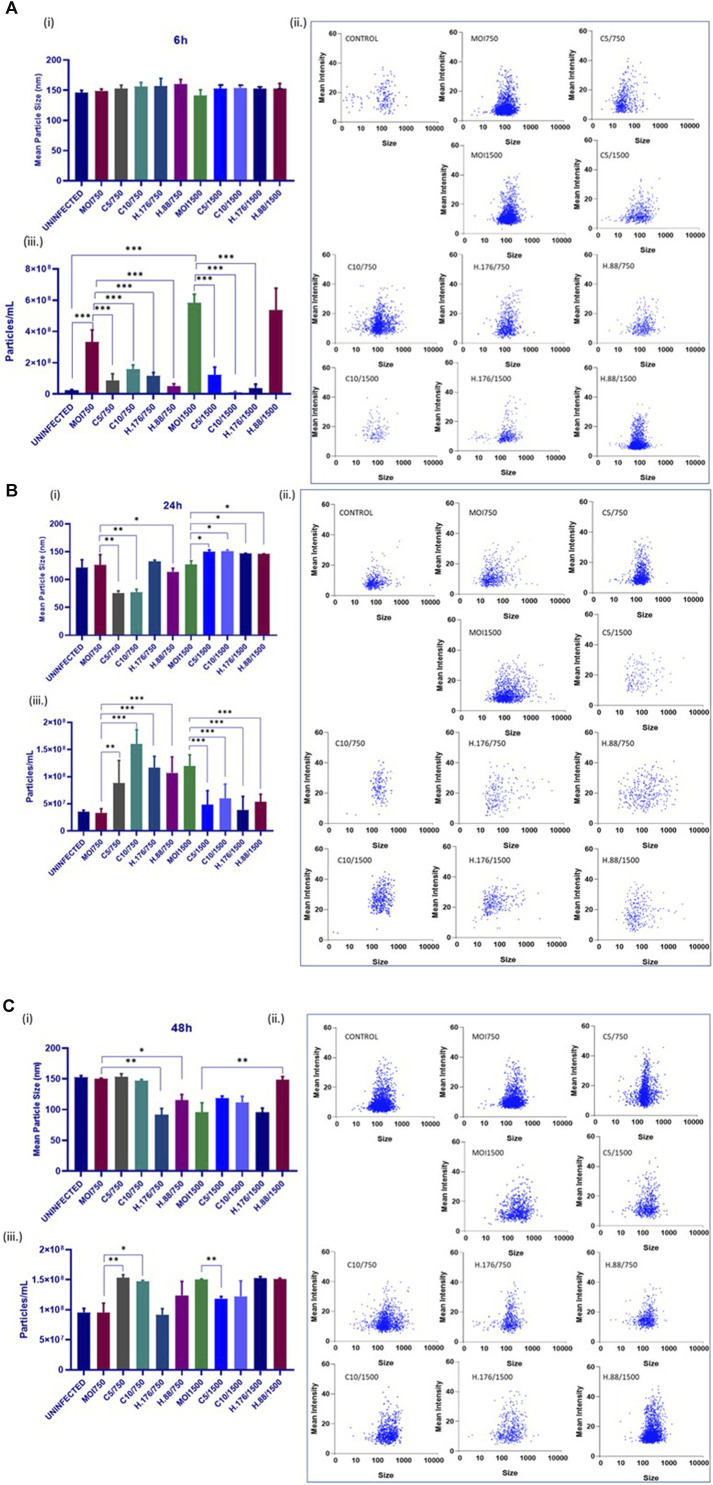
HAdV3 reduced inhibitor-treated exosome particle sizes but increased particle concentration. A549 cells were cultured in exo-free DMEM media and then treated with HAdV3-Climbazole, 5 µM (C5) and 10 µM (C10) or HAdV3-Heparin mixture 0.176 µM (H.176) and 0.88 µM (H.88) and MOIs (750 and 1500) of HAdV3 (*n* = 4). Infected cells only served as control. Zeta-particle tracking analysis showing exosome data at **(A)** 6, **(B)** 24, and **(C)** 48 h. At each time point, we determined i) mean size, ii) particle scatterplot, and iii) concentration of particle distribution after virus infection and inhibitor treatments. There were no changes in exosome particle sizes after 6 h of infection in the presence of Climbazole and Heparin but particle concentration significantly increased relative to that of untreated cells, 24 h treated cell-derived exosomes exhibited reduced particle sizes at lower concentration of inhibitors relative to untreated, however, particle concentration increased at the same concentration. Mean values and SD were derived from four independent experiments. *Denotes significance at different levels of significance compared to controls and was calculated by one-way ANOVA using GraphPad Prism, Significance levels (*p < 0.05, **0.01 and ***0.001).

The size and concentration of the total isolated particles were determined by the Particle Metrix analysis. This method allows for precise measurement of particle sizes. Samples were examined at 11 reading positions. Grubber readings were excluded to ensure only the exosome size range. There were no changes in exosome particle sizes after 6 h of infection in the presence of Climbazole and Heparin, but particle concentration significantly increased relative to that of untreated cells (Figure 4A). In addition, 24 h treated cell-derived exosomes exhibited reduced particle sizes at lower concentration of inhibitors relative to untreated (*p* > 0.001). However, exosome particle concentration increased in treatments with MOI750 and MOI1500 at 0.88 µM of Heparin after 24 and 48 h (Figures 4B, C). This suggests that the inhibitors might have a concentration-dependent effect on exosome size reduction. Notably, while Heparin had a consistent effect on exosome particle concentration, different MOIs of HAdV3 appeared to impact this parameter differently. This suggests that different inhibitory concentrations or MOIs may affect exosome release and concentration. The findings suggest a complex interplay between Climbazole, Heparin, and HAdV3 in modulating exosome size and concentration over time. Further research is needed to elucidate the underlying mechanisms behind these observations and their potential implications for viral infections and exosome biology.

### HAdV3 induced distinct effects on CD63 and Alix expression levels in A549-exosomes in the presence of Climbazole and Heparin

**FIGURE 5:**
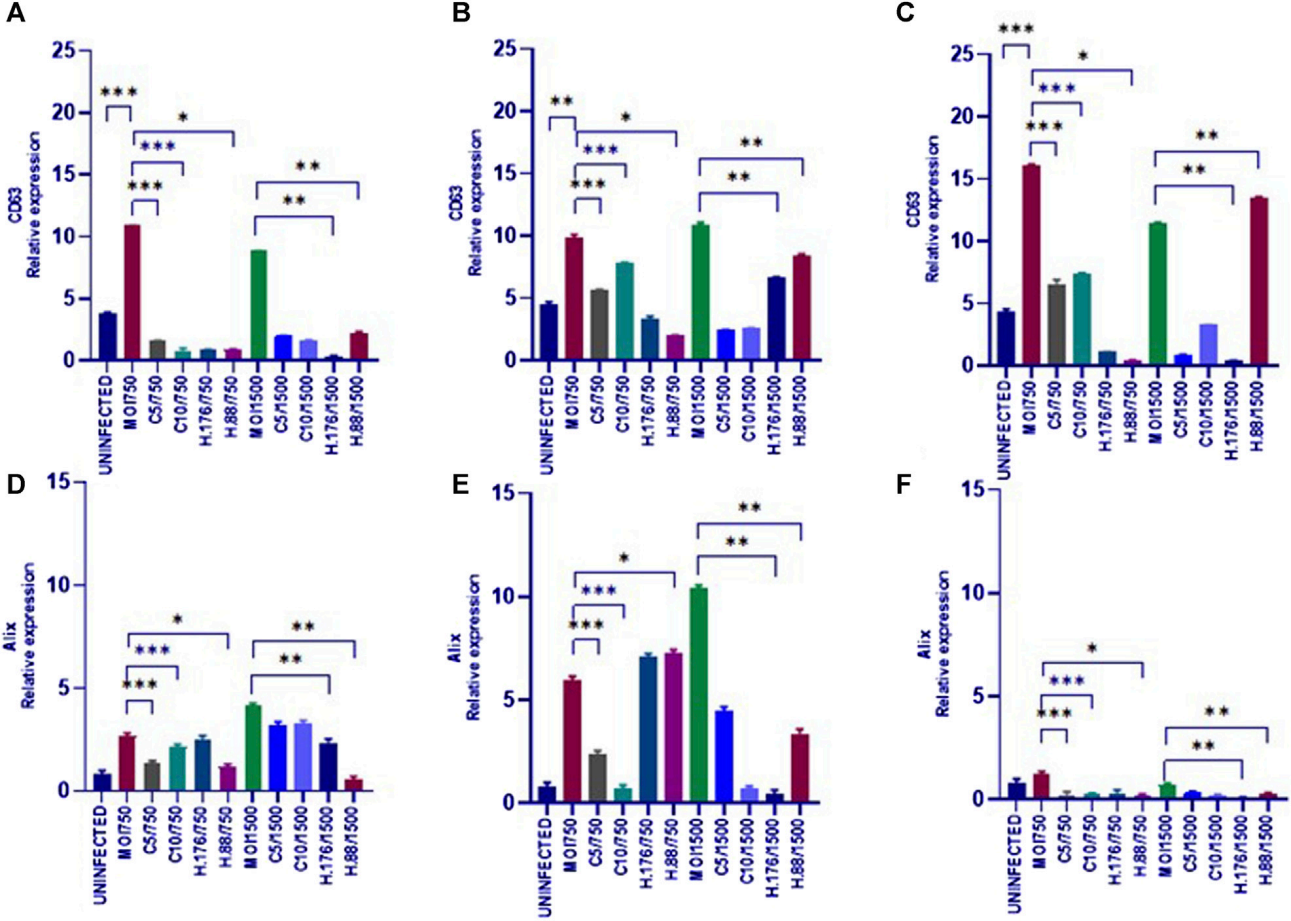
HAdV3 positively modulates CD63 and Alix in the presence of Heparin. **(A)** A549 cells were maintained overnight in exosome-free DMEM media and then treated with HAdV3-Climbazole, 5 µM (C5) and 10 µM (C10) or HAdV3-Heparin mixture 0.176 µM (H.176) and 0.88 µM (H.88) and MOIs (750 and 1500) of HAdV3 (*n* = 4). Infected cells only served as control for CD63 at **(A)** 6, **(B)** 24, and **(C)** 48 h, and for Alix at **(D)** 6, **(E)** 24, and **(F)** 48 h. HAdV3 increased CD63 and Alix levels after 24 h of infection in the presence of Climbazole and Heparin. Mean values and SD were derived from four independent experiments. *Denotes significance at different levels of significance compared to controls and was calculated by one-way ANOVA using GraphPad Prism, Significance levels (*p < 0.05, **0.01 and ***0.001). Zeta-particle tracking analysis showing exosome A.) mean size B.) total particle concentration and C.) scatterplot of particle distribution after 6 h virus infection and inhibitor treatments. Significance (*p < 0.05, **0.01 and ***0.001)*.*

To analyze the effects of HAdV3 on CD63 expression after exosome inhibition, we implemented a dot blot protein analysis. Exosome production in A549 cells was inhibited by pre-incubation with Climbazole (5 and 10 µM) and Heparin (0.76 and 0.88 µM) as reported previously (Ipinmoroti et al., 2023) and cells were further treated with HAdV3. HAdV3+Climbazole treatments had no specific effect on CD63 levels at 6 h. Notably, HAdV3 augments CD63 levels with significant percentage in HAdV3(MOI750)-Climbazole treated A549-exo at 24 and 48 h, however, HAdV3(MOI1500)+Heparin led to an increased CD63 expression at 24 h relative to the control. Similarly, Alix expression was significantly augmented in HAdV3(MOI750 and 1500)-Heparin and Climbazole treated A549-exo after 6 and 24 h relative to the control (Figures 5A–F). From these findings, it appears that different treatments involving co-incubation of HAdV3 and Climbazole and Heparin, have distinct effects on CD63 and Alix expression levels in A549-exo cells at various time points (24 and 48 h for CD63, and 6 and 24 h for Alix). In summary, the findings suggest that HAdV3, in combination with Climbazole and Heparin, has distinct effects on the expression of CD63 and Alix in A549-exo cells, with the outcomes being influenced by the HAdV3, the specific treatment, and the time point of measurement. These observations may have implications for understanding the molecular interactions and mechanisms involved in these treatments and their effects on exosome-related proteins.

### Caspase 1 and IL-1β declined in the presence of HAdV3

**FIGURE 6:**
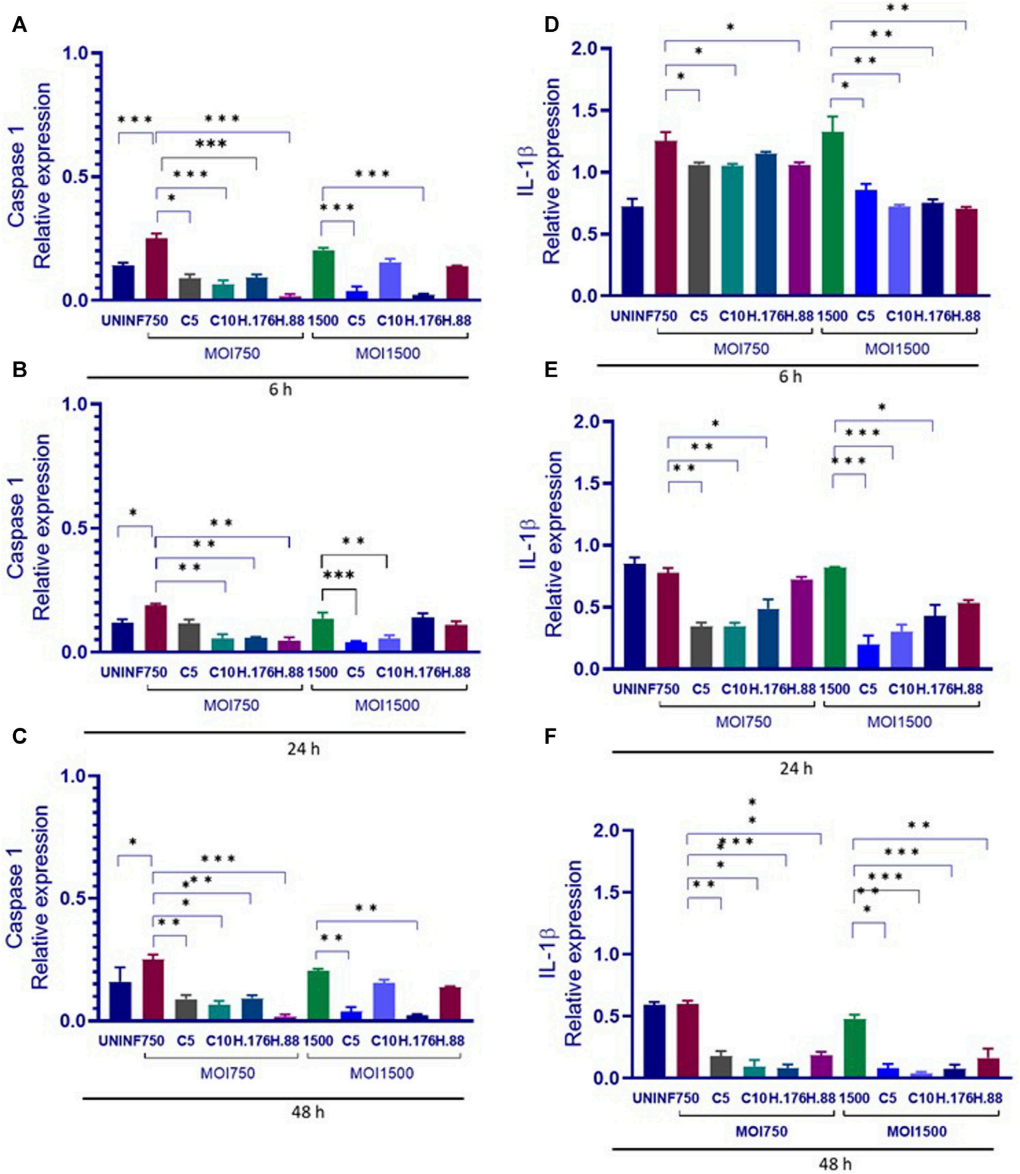
Caspase 1 and IL-1β declined in the presence of HAdV3. **(A)** A549 cells were maintained overnight in exosome-free DMEM media and then treated HAdV3-Climbazole, 5 µM (C5) and 10 µM (C10) or HAdV3-Heparin mixture 0.176 µM (H.176) and 0.88 µM (H.88) and MOIs (750 and 1500) of HAdV3 (*n* = 4). Infected cells only served as control for Caspase 1 at **(A)** 6, **(B)** 24, and **(C)** 48 h, and for IL-1β at **(D)** 6, **(E)** 24, and **(F)** 48 h. Caspase 1 level was not significantly altered, however, IL-1β level significantly increased at 6 h with HAdV3 treatment, then declined at 24 h and 48 h of infection in the presence of Climbazole and Heparin relative to uninfected. Mean values and standard deviations SD were derived from four independent experiments. *Denotes significance at different levels of significance compared to controls and was calculated by one-way ANOVA using GraphPad Prism, Significance levels (*p < 0.05, **0.01 and ***0.001).

The relationship between caspase 1 and IL-1β involves Caspase 1’s role in the processing and activation of IL-1β. Caspase 1 is an enzyme that plays a crucial role in the inflammatory response. It is responsible for cleaving pro-IL-1β into its active form, IL-1β. This cleavage step is necessary for IL-1β to become biologically active and initiate inflammatory processes. This relationship between the two molecules is a key component of the innate immune response and inflammation regulation in the body. Dysregulation of this process can lead to various inflammatory diseases and conditions. We evaluated the effect of HAdV3 infection of Caspase 1 and IL-1β expression in A549-exo treated with Climbazole and Heparin. Caspase 1 level in Climbazole and Heparin-treated A549-exosomes was not significantly altered in the presence of HAdV3 at all-time points when compared to the control (Figures 6A–C). However, IL-1β level significantly increased at 6 h with HAdV3 treatment but declined at 24 h and 48 h of infection in the presence of Climbazole and Heparin relative to control (Figures 6D–F). This suggests that the presence of Climbazole and Heparin did not significantly impact the levels of Caspase 1 in A549-exosomes when exposed to HAdV3 at 6, 24, and 48 h. However, the levels of IL-1β exhibited a distinct pattern. It significantly increased at 6 h with HAdV3 treatment but declined at 24 h and 48 h in the presence of Climbazole and Heparin when compared to uninfected conditions. This suggests that Climbazole and Heparin may influence the early release of IL-1β in response to HAdV3 infection, but do not seem to have a significant effect on Caspase 1 levels over the same time frame. Further research is needed to understand the specific mechanisms underlying these observations and their potential implications for immune responses during infection.

### Sustained HAdV3 effect on TLR7 and H2A-X expressions in A549-exo

**FIGURE 7:**
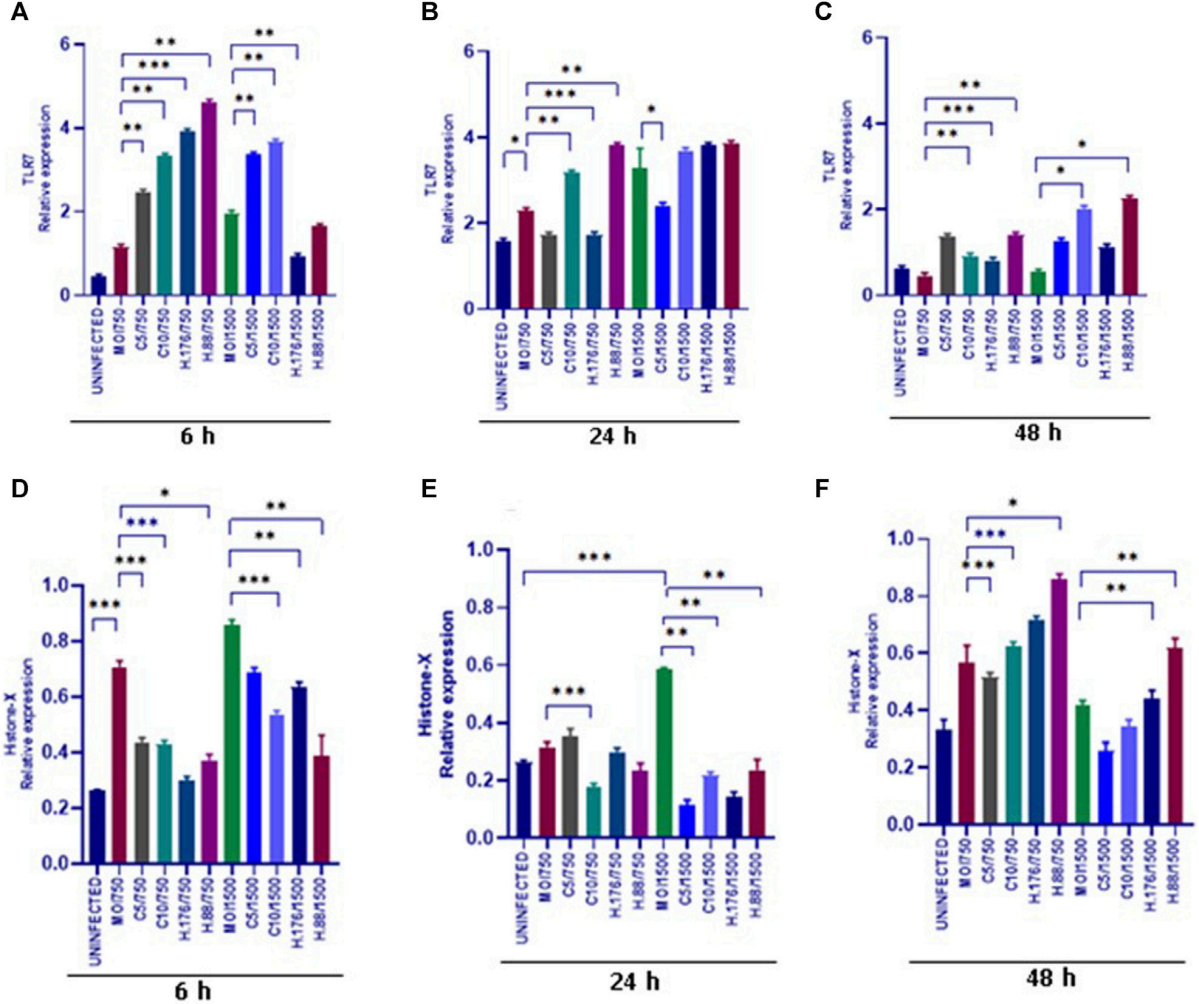
HAdV3 positively modulates TLR7 and Histone-X in the presence of Climbazole and Heparin. **(A)** A549 cells were maintained overnight in exosome-free DMEM media and then treated with HAdV3-Climbazole, 5 µM (C5) and 10 µM (C10) or HAdV3-Heparin mixture 0.176 µM (H.176) and 0.88 µM (H.88) and MOIs (750 and 1500) of HAdV3 (*n* = 4). Infected cells only served as control for TLR7 **(A)** 6, **(B)** 24, and **(C)** 48 h. HAdV3 significantly increased the expression of TLR7 and Histone-X proteins at **(D)** 6, **(E)** 24, and **(F)** 48 h of HAdV3 infection in the presence of Climbazole and Heparin, TLR7 was significantly in Heparin treated A549-exo relative to Climbazole treated A549-exo. *Denotes significance at different levels of significance compared to controls and was calculated by one-way ANOVA using GraphPad Prism, Significance levels (*p < 0.05, **0.01 and ***0.001).

We evaluated the expression of TLR7. Upregulation of TLR7 was observed in all HAdV3-Climbazole and HAdV3+Heparin treatments at all-time points compared to the control (Figures 7A–C). This indicates that the effect of HAdV3-Climbazole and HAdV3-Heparin on TLR7 expression was consistent over time. In addition, upregulation of TLR7 expression consistently across all three time points suggest that this effect is not transient but sustained over the observed period. TLR7 is known to play a role in innate immune responses, particularly in recognizing viral RNA, therefore its upregulation may indicate an enhanced antiviral response. These findings suggest a sustained immune response or cellular reaction. This upregulation suggests an involvement of TLR7 in the response to these treatments and may have implications for the immune response or cellular processes related to the presence of HAdV3. Further research is needed to fully understand the mechanisms and consequences of this upregulation. H2A-X protein expression was observed to be upregulated relative to the control treatment in response to both HAdV3(MOI1500)+Climbazole and HAdV3(MOI1500)+Heparin treatments after 6 h of treatment (Figure 7D). This suggests that these treatments have a direct or indirect impact on the expression of the H2A-X protein. The absence of upregulation at 24 h suggests that there might be a temporary delay or a different mechanism involved in the regulation of H2A-X protein expression during this specific time point. It is noteworthy that the upregulation of H2A-X protein expression was consistent with both HAdV3-Climbazole and HAdV3-Heparin treatments. This consistency could imply a common underlying mechanism or pathway through which these treatments influence H2A-X protein expression. While these findings provide valuable insights, further research is needed to understand the precise molecular mechanisms responsible for the observed upregulation of H2A-X protein expression in response to HAdV3-Climbazole and HAdV3-Heparin treatments. Additionally, the functional significance of this upregulation and its implications in the broader biological context should be explored.

### Climbazole and Heparin inhibited the expression of HAdV3 fiber

**FIGURE 8:**
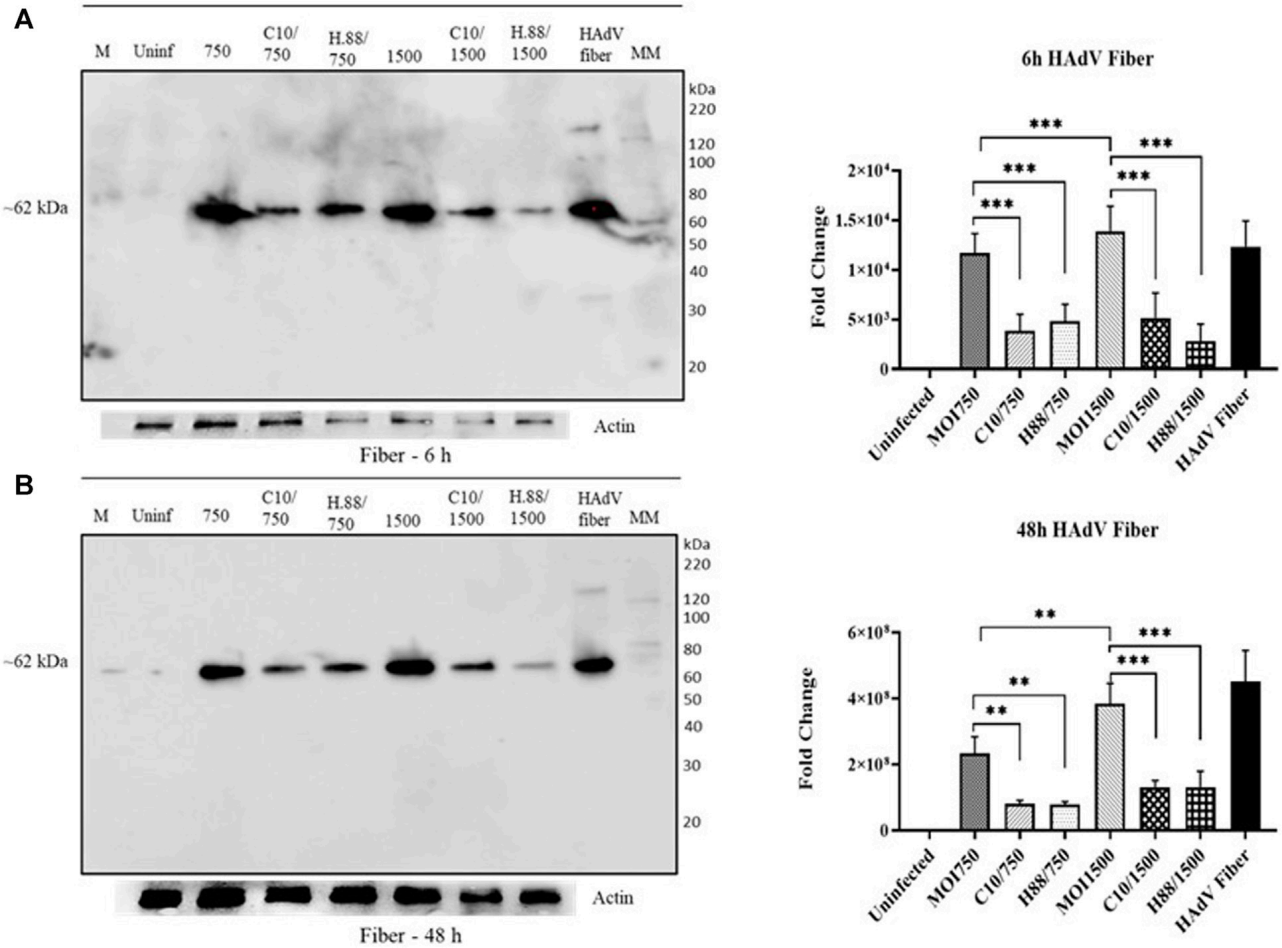
Climbazole and Heparin inhibit expression HAdV3 fiber protein. **(A)** A549 cells were maintained overnight in exosome-free DMEM media and then treated with HAdV3-Climbazole, 5 µM (C5) and 10 µM (C10) or HAdV3-Heparin mixture 0.176 µM (H.176) and 0.88 µM (H.88) and MOIs (750 and 1500) of HAdV3 (*n* = 4). Infected cells only served as control for 6, 24 and 48 h. Western blot shows fiber protein expression after **(A)** 6 h, and **(B)** 48 h infection of A549 the presence inhibitors. Mean values and SD were derived from four independent experiments. *Denotes significance at different levels of significance compared to controls and was calculated by one-way ANOVA using GraphPad Prism, Significance levels (*p < 0.05, **0.01 and ***0.001).

We further determined the effect of Climbazole and Heparin concentrations on HAdV3 entry via the expression of its fiber protein. We observed a significant decline in the expression of HAdV3 fiber in all treatments with Climbazole and Heparin in a concentration-dependent phenomenon (Figures 8A, B). The decrease in HAdV3 fiber protein expression implies that Climbazole and Heparin treatments are inhibiting the entry of HAdV3 into cells. The fiber protein is critical for the attachment of the virus to host cells. Therefore, a reduction in its expression is likely to impede the virus’s ability to infect host cells. Climbazole and Heparin may have potential antiviral properties, as they appear to interfere with the initial stages of HAdV3 infection by inhibiting viral entry. This could be significant in the context of developing treatments or strategies to combat HAdV3 infections. The findings may have clinical implications, especially if HAdV3 infections are a concern in certain medical settings. Climbazole and Heparin could potentially be explored as therapeutic agents to prevent or mitigate HAdV3 infections. While these results are promising, further research is needed to understand the exact mechanisms through which Climbazole and Heparin inhibit HAdV3 entry. Additionally, safety and efficacy studies would be required to assess the feasibility of using these compounds as antiviral agents in clinical settings.

## Discussion

This research discusses experimental results that shed light on the effects of HAdV3, Climbazole, and Heparin agents on A549 exosome (A549-exo) composition and protein expression. The choices of drugs (Climbazole and Heparin) and concentrations were based on our previous studies involving time and concentration-dependent exosome inhibitory dosage determination (Ipinmoroti et al., 2023). These experiments found that the total concentration of exosomal RNA (exoRNA) was lower initially in A549 cells treated with HAdV3 Climbazole and Heparin complexes compared to A549 cells infected with HAdV3 alone. Interestingly, despite the initial reduction in exoRNA at 6 h, the exoRNA and exoDNA content in the cells treated with HAdV3 Climbazole and Heparin complexes became slightly higher after 24 and 48 h of treatment. This indicates that the treatment might have some delayed effects on exosomal nucleic acid content. The study demonstrates changes in exoRNA and exoDNA content, exosome protein release, and the expression of specific markers and viral components in response to various treatments. These findings offer valuable insights into the interplay between viral infection and the host exosome system, potentially opening new avenues for understanding viral pathogenesis and therapeutic interventions. Exosomes are extracellular vesicles secreted by cells, playing essential roles in intercellular communication and disease progression (Ipinmoroti et al., 2021; Ipinmoroti et al., 2022). Viruses, such as HAdV3, have been shown to interact with exosomes during infection, altering their composition and cargo (Ipinmoroti et al., 2021). This research perspective examines the impact of HAdV3, Climbazole, and Heparin complexes on A549-Exo composition and protein expression, shedding light on the intricate dynamics of viral infection and host responses.

The initial observation of lower total exoRNA concentration in A549-Exo treated with HAdV3-Climbazole and Heparin complexes, compared to HAdV3-infected A549-Exo, suggests a potential influence of these complexes on exoRNA dynamics. These findings suggest that while initial viral infection may lead to reduced exoRNA levels, prolonged exposure to Climbazole and Heparin could modulate exosome cargo, potentially impacting host responses and viral replication.

A significant reduction in exosome protein release was observed in HAdV3-treated exosomes when exposed to Climbazole and Heparin, suggesting the potential role of these complexes in modulating exosome secretion. While total exosome protein levels remained similar to uninfected exosomes after 24 and 48 h, a slight reduction was noted after 48 h of treatment. This suggests that Climbazole and Heparin may affect the regulation of exosome protein release over time.

The study observed a significant reduction in the release of exosomal proteins in cells treated with HAdV3 in the presence of Climbazole and Heparin. This suggests that these compounds can influence the secretion of exosomes from infected cells. Total exosome protein levels were similar, but slightly reduced after 48 h. While the total exosome protein levels were similar to uninfected exosomes after 24 and 48 h of treatment, there was a slight reduction in protein levels after 48 h. This implies that the effects of the treatment on exosomal protein levels might become more pronounced with longer exposure.

The study also investigated the expression of specific exosome markers, such as CD63, Alix, and TLR7, as well as the viral component HAdV3 fiber. HAdV3 had no specific effect on CD63 levels at 6 h, but a significant increase was observed in HAdV3(MOI750)+Climbazole-treated A549-Exo at 24 and 48 h.

Similarly, HAdV3(MOI1500)+Heparin led to increased CD63 expression at 24 h. These findings suggest that Climbazole and Heparin may potentiate CD63 expression, potentially influencing exosome functions. The treatment with HAdV3 and Climbazole did not have a specific effect on the CD63 protein levels at the 6-hour time point.

Remarkably, the combination of HAdV3 and Climbazole increased CD63 expression significantly at 24 and 48 h. CD63 is a tetraspanin protein often associated with exosomes, suggesting that this combination might affect exosome-related processes (Ipinmoroti et al., 2021). When Heparin was used in combination with HAdV3, it led to increased CD63 expression at the 24-hour time point. This also indicates that Heparin can modulate exosome-related protein expression.

The upregulation of Alix expression was significant in HAdV3(MOI750 and 1500)+Heparin and Climbazole treated A549-Exo after 6 and 24 h. Alix expression augmented with HAdV3+Heparin and Climbazole at 6 and 24 h. The experiment showed an upregulation of Alix expression in cells treated with HAdV3 in combination with Heparin and Climbazole at the 6 and 24 h time points. Alix is another protein often associated with exosome formation and cargo loading (Ipinmoroti et al., 2021). Climbazole has been shown to decrease the levels of both Alix and Rab27a significantly in human prostate cancer epithelial metastatic cell line PC-3 (Datta et al., 2018). This indicates a potential role for these complexes in modulating Alix-associated processes within exosomes. We have previously demonstrated that Climbazole concentrations (5 and 10 µM) inhibited exosome particle concentration and effectively downregulated CD63, Rab27a, and TSG101 expressions in A549 cells (Ipinmoroti et al., 2023). Another study showed that Climbazole, the lead compound of 4580 screened compounds effectively inhibited exosome release by CD63-GFP-expressing C4-2B cells (Liu and Su, 2019). Protein TLR7 was upregulated at various time points (6, 24, and 48 h) after treatment with the described compounds. TLR7 is involved in immune responses, and its upregulation may indicate a response to viral infection and treatment. Notably, all treatments with Climbazole and Heparin led to a significant decline in the expression of HAdV3 fiber in a concentration-dependent manner.

In this study, cells infected with Human Adenovirus type 3 (HAdV3) and treated with Climbazole, and Heparin demonstrated reduction in exosomal levels of Caspase 1 and interleukin-1 beta (IL-1β) over time following this treatment. This finding suggests that Climbazole and Heparin could interfere with the inflammatory response typically observed in HAdV3 infection. Caspase 1 is crucial for the maturation and release of IL-1β, a key pro-inflammatory cytokine. The reduced levels of Caspase 1 and IL-1β in exosomes suggest that Climbazole and Heparin might be altering the exosomal packaging and secretion processes. Since Caspase 1 is involved in the cleavage of pro-IL-1β to its active form, its reduction in exosomes could mean less active IL-1β is being released by infected cells, potentially leading to a dampened inflammatory response. The reduced levels of these molecules could indicate an inhibited inflammatory response. During HAdV3 infection, the inflammatory response is a key aspect of the body’s defense but can also contribute to disease pathology. Therefore, reducing excessive inflammation could be beneficial in managing symptoms and potentially reducing tissue damage. This result suggests significant implications for treating HAdV3 infections. If Climbazole and Heparin can effectively reduce harmful inflammation without compromising viral clearance, they could represent a novel therapeutic strategy.

The expression of HAdV3 fiber, a viral protein, was significantly reduced in all treatments with Climbazole and Heparin. This reduction was concentration-dependent. This suggests that these compounds may interfere with the expression of viral proteins. The azole family has been implicated in their role in modulating exosome biogenesis (Datta et al., 2018; Liu and Su, 2019; Damme et al., 2021; Ipinmoroti et al., 2023). A study has demonstrated itraconazole treatment resulted in significant viral yield reduction as measured in both Caco‐2 cells and VeroE6‐eGFP cells (Damme et al., 2021). There is obvious reciprocal interaction between exosome biogenesis and virus replication. Viruses utilize various endocytic pathways like clathrin-dependent endocytosis, micropinocytosis, and lipid raft-dependent endocytosis for entry into host cells (Mardi et al., 2023). This is akin to the pathways used in exosome biogenesis and release, suggesting shared cellular machinery. The process of virus entry and exosome formation both involve complex interactions with the host cell’s membrane. For instance, the involvement of GTPase activity in dynamin, which is crucial in endocytic vesicle fission, is a common feature in both viral entry and exosome release (Mardi et al., 2023). This indicates that viruses might exploit the same signaling pathways that regulate exosome biogenesis. Both processes involve intricate vesicle trafficking and fusion mechanisms within the host cell (Crenshaw et al., 2019b; Ipinmoroti et al., 2021; Mardi et al., 2023). The mechanisms that govern endosomal trafficking and fusion with target membranes are critical in determining the fate of both exosomes and viruses within cells.

In summary, these experimental results indicate that the combination of HAdV3 with Climbazole and Heparin has complex effects on exosomal content, protein expression, and viral protein levels in A549 cells. These findings have implications for understanding the interplay between viral infection and exosome biology and may have relevance for antiviral strategies and therapeutic interventions. However, further research is likely needed to fully elucidate the mechanisms underlying these observations and their broader significance. Future investigations might involve conducting *in vivo* experiments to corroborate our *in vitro* results. This step is crucial for validating the findings and evaluating the potential therapeutic effectiveness and safety of the inhibitors in animal models. Subsequent studies could extend their scope to include long-term effects of these inhibitors on infections and *vice versa*. Examining the prolonged impact of HAdV3 infection and inhibitor treatment on exosome biogenesis, cargo composition, and cellular health and function would provide a comprehensive understanding. This observation suggests that these complexes may interfere with viral attachment and entry. The experimental results presented in this study highlight the complex interplay between HAdV3, Climbazole, and Heparin complexes in A549-Exo composition, protein release, and marker expression. These findings open new avenues for investigating the role of exosomes in viral infections and potential therapeutic strategies targeting exosome-mediated viral dissemination and immune responses. Further research is warranted to elucidate the underlying mechanisms driving these observed changes and their implications for viral pathogenesis.

## Data Availability

The original contributions presented in the study are included in the article/supplementary material, further inquiries can be directed to the corresponding author.
